# Evaluating the Risk of Epidemic Thunderstorm Asthma: Lessons from Australia

**DOI:** 10.3390/ijerph16050837

**Published:** 2019-03-07

**Authors:** Sharon L. Campbell, Paul D. Fox-Hughes, Penelope J. Jones, Tomas A. Remenyi, Kate Chappell, Christopher J. White, Fay H. Johnston

**Affiliations:** 1 Menzies Institute for Medical Research, University of Tasmania, 1 Liverpool St, Hobart, TAS 7000, Australia; sharon.campbell@utas.edu.au (S.L.C.); penelope.jones@utas.edu.au (P.J.J.); kate.chappell@utas.edu.au (K.C.); 2 Public Health Services, Department of Health (Tasmania), 25 Argyle St, Hobart, TAS 7000, Australia; 3 Bureau of Meteorology, GPO Box 727, Hobart, Tasmania 7001, Australia; paul.fox-hughes@bom.gov.au; 4 Antarctic Climate and Ecosystems Cooperative Research Centre, University of Tasmania, 20 Castray Esplanade, Hobart, TAS 7000, Australia; tom.remenyi@utas.edu.au (T.A.R.); chris.white@utas.edu.au (C.J.W.); 5 School of Engineering, University of Tasmania, Private Bag 65, Hobart, TAS 7001, Australia; 6 Department of Civil and Environmental Engineering, University of Strathclyde, James Weir Building, 75 Montrose Street, Glasgow G1 1XJ, UK

**Keywords:** asthma, thunderstorm, public health, risk, hazard

## Abstract

Epidemic thunderstorm asthma (ETA) is an emerging public health threat in Australia, highlighted by the 2016 event in Melbourne, Victoria, that overwhelmed health services and caused loss of life. However, there is limited understanding of the regional variations in risk. We evaluated the public health risk of ETA in the nearby state of Tasmania by quantifying the frequency of potential ETA episodes and applying a standardized natural disaster risk assessment framework. Using a case–control approach, we analyzed emergency presentations in Tasmania’s public hospitals from 2002 to 2017. Cases were defined as days when asthma presentations exceeded four standard deviations from the mean, and controls as days when asthma presentations were less than one standard deviation from the mean. Four controls were randomly selected for each case. Independently, a meteorologist identified the dates of potential high-risk thunderstorm events. No case days coincided with thunderstorms during the study period. ETA was assessed as a very low risk to the Tasmanian population, with these findings informing risk prioritization and resource allocation. This approach may be scaled and applied in other settings to determine local ETA risk. Furthermore, the identification of hazards using this method allows for critical analysis of existing public health systems.

## 1. Introduction

Epidemic thunderstorm asthma (ETA) is seen as an emerging public health threat in Australia and other parts of the world, creating the need to develop a sustained level of community resilience and preparedness in affected regions [1].

**Figure 1:**
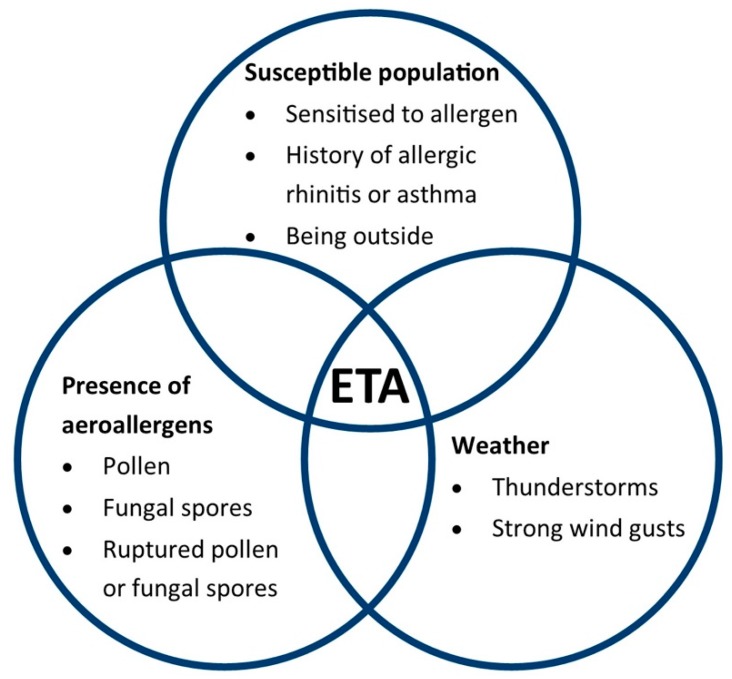
Intersection between aeroallergens, specific weather conditions, and a susceptible population, giving rise to a potential epidemic thunderstorm asthma (ETA) event (adapted from [5]).

The mechanism of ETA, described in detail elsewhere [2,3,4], involves the concurrent presence of (a) aeroallergens (e.g., pollen, ruptured pollen, or fungal spores), (b) specific weather conditions (thunderstorms and strong wind gusts), and (c) a susceptible population group who are sensitized to the aeroallergen and have a history of allergic rhinitis or asthma [5] (see Figure 1).

Due to the requirement for the concurrent presence of these three components, ETA events are very rare, having been recorded on only 10 occasions in southeastern Australia: seven times in Melbourne (Victoria), and once in Canberra (Australian Capital Territory), Newcastle (New South Wales), and Wagga Wagga (New South Wales), respectively [5]. Twelve notable events have been recorded in other areas of the world [5], with a number of additional studies also showing a positive association between asthma presentations and thunderstorm events [6]. These may potentially indicate further previously undetected ETA events.

Each of the Australian events has caused an increase in emergency department presentations and/or admissions [5]. The most striking of these events occurred in Melbourne, Victoria, on 21 November 2016, which caused an exceptional level of demand on ambulance and hospital services and a number of deaths [1,7]. This event was widely publicized, with considerable media commentary provided about the community impact, emergency response, and steps needed to reduce the impact of this event in the future [8,9,10]. In response, a substantial research investment in forecasting and predicting these events was provided by the Victorian Government [11]. In addition to research assessing the public health impact, this rare event was examined, in detail, from a meteorological perspective [12].

The thunderstorm complex associated with the 2016 Melbourne event moved south and crossed Bass Strait, passing over northern Tasmania (including the population centers of Burnie and Launceston). Investigations at the time showed no increase in ambulance or emergency department activity in those Tasmanian regions, in contrast to the severe health outcomes in Melbourne. However, the severity of the Melbourne event—and its proximity to Tasmania—prompted significant local concern about the potential for an ETA event to occur in the state. This was highlighted by the extensive traditional and social media response to a subsequent thunderstorm asthma warning issued by the Tasmanian Department of Health and Human Services one year later [13,14,15]. In this context, public health policymakers identified a gap in understanding how and where these events occur in Tasmania, and to what degree they pose a risk. This study seeks to redress this gap and create an assessment methodology that can be applied across other at-risk regions.

### 1.1. Study Location

Tasmania is the only island state of Australia. Melbourne, Victoria, lies approximately 300 km north of the state’s north coast, separated by Bass Strait. The majority of the Tasmanian population resides in a regional or remote classified area [16]. The state’s total population in 2016 was 510,000, with the majority of the population residing in one of three major centers: Hobart (population 204,000), Launceston (population 84,150), or Burnie–Devonport (population 70,000) [17].

Tasmania has four major public hospitals located in the most densely populated regions of the state—one located in Hobart (Royal Hobart Hospital); one in Launceston (Launceston General Hospital); and two in the Burnie–Devonport region (the Mersey Community Hospital and the North West Regional Hospital). Each of these hospitals has an emergency department.

### 1.2. Thunderstorms in Tasmania

In contrast to continental southeastern Australia, Tasmania has a more maritime and temperate climate, with less frequent thunderstorms overall [18]. In particular, Tasmania experiences fewer ‘dry microburst’ thunderstorms that can potentially generate strong wind gusts [19]. Furthermore, the Tasmanian topography and predominantly westerly flowing air mass influence the occurrence and location of thunderstorms across the state. More storms are reported in the west and north of the state as air is forced to ascend when these air masses encounter the Tasmanian landmass, and this ascent is conducive to the development of thunderstorms under some conditions. In contrast, these same westerly winds descend along the contour of the landscape into southeastern Tasmania, rendering thunderstorms less common in this region [18,20].

### 1.3. Pollen in Tasmania

**Figure 2:**
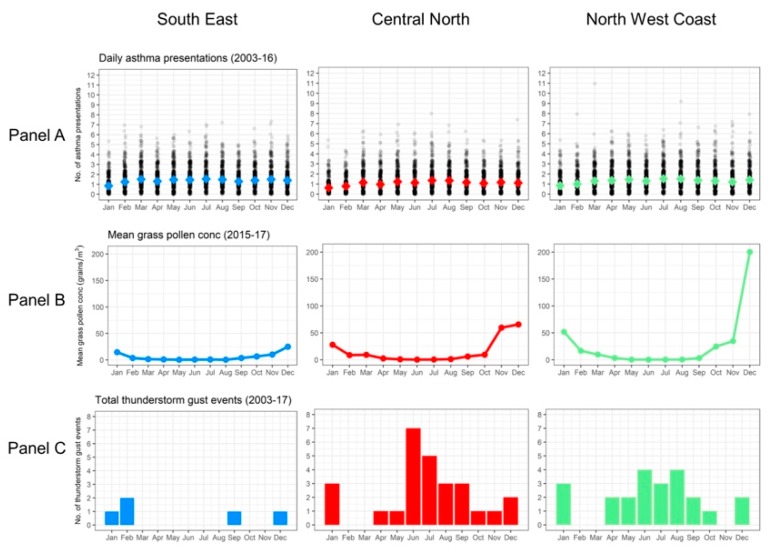
Daily asthma presentations (Panel A), mean grass pollen concentrations (Panel B), and total thunderstorm asthma gust events (Panel C) for each of the relevant Tasmania forecast districts (South East, Central North, and North West Coast).

**Figure 3:**
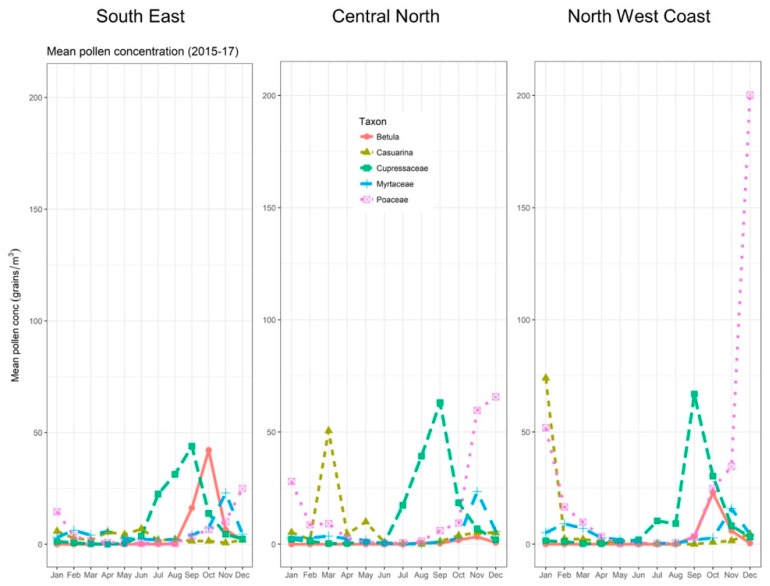
Pollen counts for each relevant Tasmanian forecast district showing the five major taxa.

With respect to pollen abundance, there are both similarities and differences between Tasmania and mainland southeastern Australia that are relevant for understanding ETA risk. Grass pollen—and, in particular, rye grass pollen—is the major aeroallergen implicated in previous Australian thunderstorm asthma events [3,5,21]. Although levels are typically lower than in mainland southeastern Australia [22], grass pollen is prevalent across Tasmania during the peak season of November to January [23,24] (see Figure 2, Panel B). As rye grass is an important pasture grass species [25], a significant proportion of this is highly likely to be rye grass-derived. Beyond grass pollen, Tasmania also experiences seasonally high loads of several other aeroallergens, including *Betula* (birch), Cupressaceae (cypress), and *Plantago* (plantain) [23,24]. These have peak seasons that are different from peak grass pollen season. Overall, this means Tasmania experiences generally high pollen conditions—though not necessarily high grass pollen concentrations—from approximately July through to January [24] (see Figure 3).

### 1.4. Natural Disaster Risk Assessment in the Tasmanian Context

Tasmania’s natural disaster risk is assessed using the Tasmanian State Natural Disaster Risk Assessment (TSNDRA) framework that aims to “produce a state-wide priority natural hazard risk assessment, in accordance with the relevant International and Australian standards” [26]. This framework is consistent with the 2015 National Emergency Risk Assessment Guidelines (NERAG), the Australian standard for natural disaster risk assessment [27], and complies with a number of relevant risk management standards [26]. Similar frameworks exist globally [28,29].

The TSNDRA framework was most recently updated in 2016, assessing state-wide natural disaster risks for the following hazards: bushfire, flood, severe storm, landslide, tsunami, earthquake, heatwave, coastal inundation, and pandemic influenza [26].

TSNDRA uses five ‘impact sectors’ to determine the overall risk for each hazard. These sectors cover a range of consequences across a broad spectrum of outcomes, including ‘People’, ‘Economic’, ‘Environmental’, ‘Public administration’, and ‘Social setting’. For more information on these sectors and how they translate to the Tasmanian context, see Supplementary Materials (Natural disaster risk assessment in Tasmania).

To assess a hazard’s overall risk level, the likelihood and consequences of the risk are ascertained for each sector and plotted on a risk matrix. The confidence of each of these plots is then assessed based on available evidence. Finally, a priority level is set, based on the risk level and confidence associated with that risk.

### 1.5. Research Aim

The aims of this study were to (a) understand the history of ETA events in Tasmania (and determine if any undetected ETA events had occurred in the study period), and (b) apply these results to the TSNDRA framework and determine the risk of these events, specifically the public health risk, in the Tasmanian context. This will determine the effectiveness of applying this methodology to other regions at risk of ETA events.

## 2. Materials and Methods

### 2.1. Study Design

In order to understand the history of thunderstorm asthma events in Tasmania, we adapted the case–control design used by Marks et al. [3], in which the unit of analysis is a day rather than an individual person. Using this approach, Marks et al. discovered a number of previously undetected ETA events in central New South Wales, in cities with similar-sized populations to Tasmania’s major centers. This demonstrates the capacity of this approach to detect ETA events in analogous demographic contexts. Here, we employed a similar study design, modified to accommodate the different climatic and geographical setting of Tasmania. We used the results of this study to assess risk in the Tasmanian context using the TSNDRA framework.

### 2.2. Health Outcomes

We obtained emergency department (ED) presentation data for public hospitals in Tasmania from the Tasmanian Health Service (THS) for the Royal Hobart Hospital, Launceston General Hospital, Mersey Community Hospital, and the North West Regional Hospital. Due to the small size and close proximity of the two hospitals in the northwest region, we combined data from the Mersey Community Hospital and North West Regional Hospital to represent the entire northwest. Data were extracted for the period from 20 December 2002 to 30 June 2017.

Presentations were identified where International Classification of Disease (ICD) codes for asthma were given as the primary diagnosis (J45, J45.0, J45.1, J45.8, J45.9, and J46). We estimated expected daily attendances for each region using a log linear model. Modeling included accounting for linear, quadratic, and cubic time trends (including overall time trends), seasonal factors, and day of the week effects. Case days were identified when the daily presentations for asthma exceeded four standard deviations (SD) from the expected value. Control days were randomly selected from all dates where the number of ICD-coded asthma presentations were less than one standard deviation from the expected value. A sensitivity analysis also examined case days where daily presentations for asthma exceeded five standard deviations. For each case day, four times the number of control days were generated. To account for potential differences in coding and to provide a more sensitive analysis, we also separately analyzed ICD-coded presentations for wheezing (R06.2) and dyspnea (R06.0). Specifics of each case (for example gender, age, type of asthma) were not considered in this analysis, as the total number of cases was the variable of interest.

### 2.3. Meteorological Data

**Figure 4:**
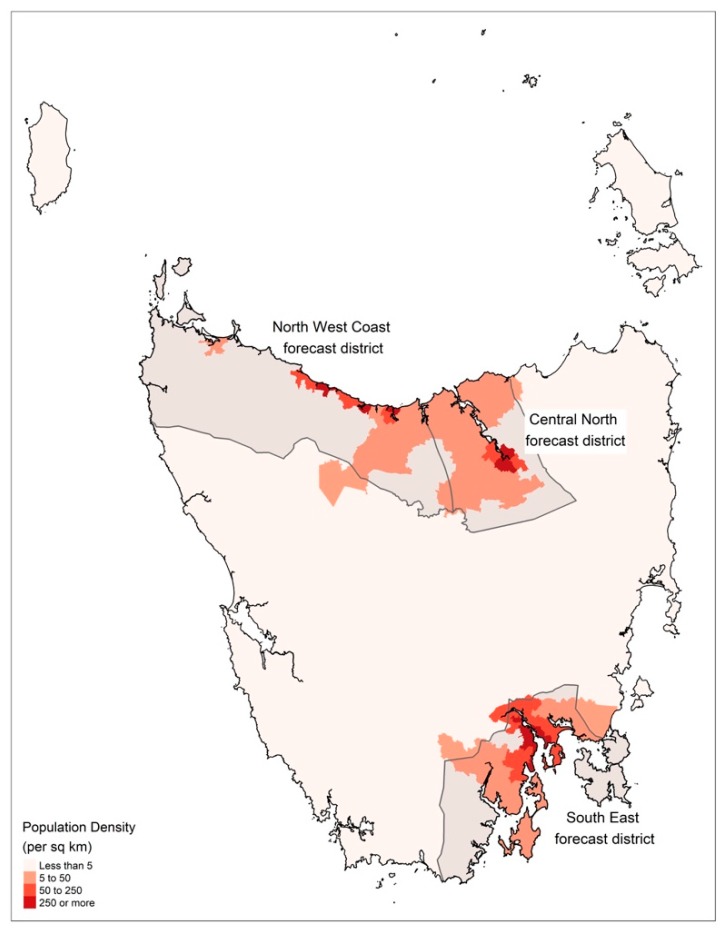
Population density in Tasmania and Bureau of Meteorology (BoM) forecast districts used in this study.

Weather observation data were obtained from the Global Position and Tracking System (GPATS) and Bureau of Meteorology (BoM) automatic weather stations (AWS). We combined lightning data from GPATS and wind data from AWS to identify likely thunderstorm gust events close to populated regions of Tasmania. Sixteen AWS were identified in the three BoM forecasts districts (South East, Central North, and North West Coast) that most closely matched the major population centers feeding the four public hospitals (see Figure 4).

The topography of Tasmania is vastly different to that of inland New South Wales, with Tasmania mostly displaying a rugged landscape of hills and valleys compared to the relatively flat plains of central New South Wales. To reflect this difference, the radius of influence of a thunderstorm gust event on a population center used in Marks et al. [3] (80 km) was reduced. Here, we identified a thunderstorm gust event if (1) a lightning strike occurred within a 0.1° rectangle (approximately 10 km) of an AWS location, and (2) a wind gust in excess of 60 km/h was recorded by the AWS within 10 min of this lightning strike. We also employed a sensitivity test to increase the strike range to within a 0.2° rectangle (approximately 20 km) and increase the time differential to 20 min, while keeping the gust strength at 60 km/h.

The list of combined asthma case and control days was given to the meteorologist, who was blinded to which days were case or control. The meteorologist then compared these days to the identified thunderstorm gust days to determine any overlap.

### 2.4. Risk Assessment

Using the TSNDRA framework [26], we analyzed the likelihood and consequence for each sector to determine the combined overall risk. For consequence levels, results of the Tasmania case–control ETA event study informed the ‘People’ sector. Learnings from the Melbourne 2016 ETA event [1,7] and other post-disaster research [30,31,32] informed the remaining sectors. For more information, see Supplementary Materials (Natural disaster risk assessment in Tasmania).

## 3. Results

### 3.1. Health Outcomes and Meteorological Data

Daily emergency department presentations for asthma for the three major population centers, as well as for the state as a whole, were analyzed (see Figure 2, Panel A). For this analysis only, dates in 2002 and 2017 were eliminated to allow for complete years of analysis (i.e., 1 January 2003 to 31 December 2016).

In Tasmania, asthma presentations remained relatively steady throughout the year and across regions, with the coldest months of July and August demonstrating the highest rate of presentations. The lowest rates for all regions occurred in January, which is the warmest month. This is generally consistent with the global pattern of seasonality in asthma [33,34,35,36].

A total of 5307 days and 2,107,594 presentations were analyzed for the whole study period. We found 19,979 asthma presentations across all regions and identified asthma case days in all regions. The total number of presentations, mean number of daily presentations, maximum number of daily presentations, and the number of case and control days for each hospital are presented in Table 1.

We also found ICD-coded presentations for wheezing and dyspnea in all regions. However, only 462 cases of wheezing and 2562 cases of dyspnea were observed across the whole study period. Given the low number of these cases, wheezing and dyspnea presentations were not analyzed further.

Figure 2, Panel C shows the total number of days per month during the study period where thunderstorm gust events were identified for each BoM forecast district. Thunderstorm gust events were rare in the South East forecast district all year round. Events were more common in the Central North and North West Coast forecast districts, with the majority occurring in the winter period.

There was no overlap between days where daily asthma presentations exceeded four SDs (case days) and thunderstorm gust events. Only one control day was identified in the South East district that overlapped with an identified thunderstorm gust event, and none in the other two districts. On this basis, no ETA events were identified in Tasmania during the study period.

### 3.2. Risk Assessment

Table S1 (see Supplementary Materials) shows the consequence categories for each sector using the TSNDRA framework, mapped against the likely outcomes of an ETA event in Tasmania and the evidence for these outcomes. We assigned a consequence rating of ‘insignificant’ across all sectors.

Based on the Tasmanian case–control ETA event study, we determined the likelihood of an event occurring in Tasmania was rare (occurring once every 100–1000 years) to very rare (occurring once every 1000–10,000 years) [26].

**Figure 5:**
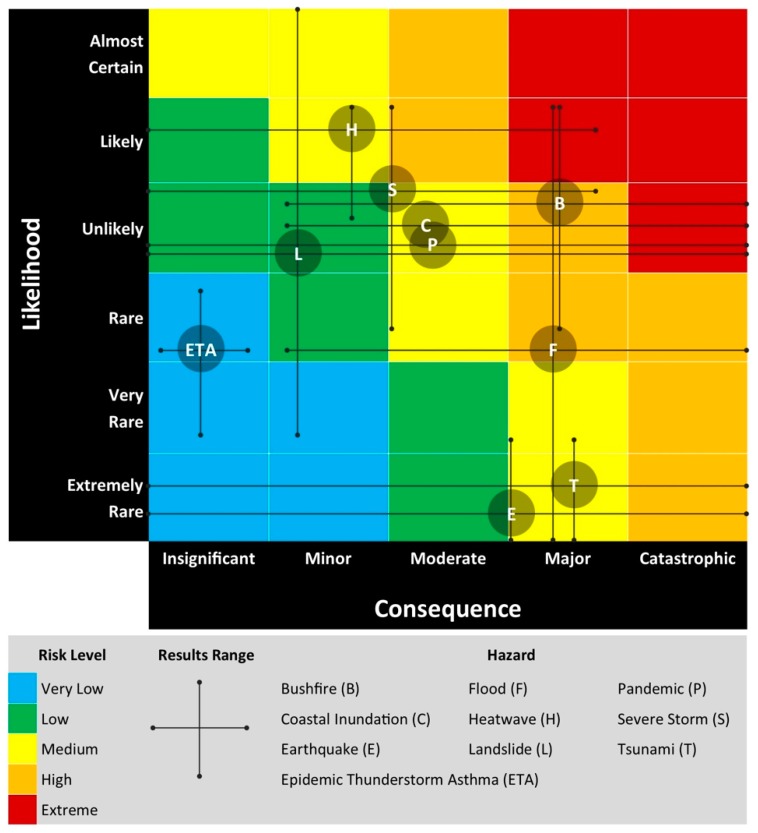
Summary of the risk level posed by each hazard as assessed in Tasmanian State Natural Disaster Risk Assessment (TSNDRA) 2016 [26], adapted to include ETA risk as determined by this study.

Using the TSNDRA framework and combining the consequence and likelihood outcomes, we determined ETA events have an overall risk rating of ‘very low’. Confidence in this result is high. This hazard is the lowest-ranked risk across all nine hazards examined in the TSNDRA framework [26] (see Figure 5). For each of the hazards in this Figure, the results range describes the consequence and likelihood across all sectors, derived from a stakeholder consultation process.

In specifically examining the public health risk of ETA events, we determined a risk level of ‘very low’ for both the ‘People’ and ‘Social setting’ sectors, and ‘insignificant’ for the other three sectors (Economic, Environment and Public Administration). Across all sectors, ETA events ranked in the lowest level of risk when compared to all natural hazards examined within TSNDRA 2016 [26].

## 4. Discussion

The results show that there were no ETA events in Tasmania during the 14.5-year study period. Using the TSNDRA framework, ETA events were categorized as a very low public health risk and a very low risk overall. Furthermore, we found that thunderstorm gust events are uncommon in the more densely populated southeastern region of Tasmania compared to the less densely populated north and northwest regions of the state, therefore reducing the likelihood of these events happening over a population center. In addition, the north of Tasmania experiences thunderstorm gust events most commonly in winter, outside the high grass pollen season.

This study uses a previously published methodology [3], adapted for Tasmania’s geography and topography. Blinding of the meteorologist to epidemic case days ensured unbiased interpretation of results. Application of a standardized risk assessment framework for natural disasters, as exists in other locations [28,29,37], makes this a robust and globally transferable model against which to measure this risk. Furthermore, this method can be easily scaled to allow investigation across variety of spatial locations, making it applicable for both smaller and larger regions.

The results of this study rely on the somewhat limited understanding of the underlying science of ETA events. While coincident high grass pollen and thunderstorm gust events are incorporated in the working theory for these event triggers, the actual mechanism for ETA events has not been conclusively proven [5]. It is possible that another as yet undiscovered mechanism has a contribution to ETA events, and this factor (or combination of factors) is not currently incorporated into the methodology.

In this study, pollen data were not examined in detail. As the aim of this study was to determine if any undetected ETA events had occurred in the study period, analysis of pollen data was not required. While pollen data exists for some areas of Tasmania [23,24], the available data does not cover the complete 14.5-year period of this study, nor for all regions of the state examined in this research. If ETA events had been detected in this study, it may have been of value to examine any available pollen data related to these events. Given a focus on the identification of epidemic thunderstorm asthma, this study does not examine the interaction between the prevalence of asthma cases and other air quality variables, such as particulate matter, wind speed, temperature, or humidity. Similarly, the impact of thunderstorms on other forms of illness or injury was not examined.

The study covers a period notably longer than other similar case–control studies examining thunderstorm events and health outcomes [3,38,39]. This allows for a more thorough examination of the temporal influences and trends impacting the result. Examining thunderstorm gust event data over this extended period has allowed for observations on frequency and timing of these events across the regions, which has further contributed to understanding risk levels.

By understanding the public health and overall state-wide risk of ETA events, policymakers are now able to determine the most appropriate local policy response to this threat, especially in a setting of finite resources and multiple and competing risk priorities. With a risk rating of ‘very low’, placing a low priority on addressing the risk of ETA events in Tasmania may be seen as a valid course of action. However, analysis of such events allows policymakers to examine the robustness of existing public health and emergency response systems when exposed to similar threats, from both known and unknown sources.

In Tasmania, asthma is a serious and common health condition, with over 12% of the population currently affected [40], and over 25% reporting they have been diagnosed with asthma at some point in their lives [41]. Nineteen people died from asthma in Tasmania in 2016 [42], compared to nine people in the Melbourne ETA event [1]. Upskilling the community in asthma management more generally is likely to have a sustainable and long-term impact and reduce the burden of this disease both within the context of an ETA event and more broadly across the community.

The contribution of environmental factors to higher-than-average daily asthma presentations is not fully understood, and further research is needed in the Tasmanian context. Potential contributors may include air pollution, weather extremes (including wind, rainfall, or temperature), high aeroallergen levels, or circulation of respiratory viruses such as colds or influenza in the community, influencing winter peaks.

The impact of climate change on the risk of ETA events is yet to be fully assessed and understood. While it is likely that pollen seasons will increase in length due to a warming climate [43,44], potential changes to the frequency and/or severity of thunderstorms are more difficult to ascertain [45]. While thunderstorms and other similar weather patterns are likely to increase in severity and frequency as a result of a warming climate [46,47], increased atmospheric stability may result in a slight decline in conditions favorable for lightning strike in Tasmania [48]. Further research in this area is required.

## 5. Conclusions

In conclusion, no ETA events were identified during the 14.5-year study period, and an examination of weather and pollen data against a standardized risk assessment framework suggests that the coincidence of thunderstorm gust events with a high rye grass pollen season occurring close to a susceptible population is very low in Tasmania. Using a similar methodology and appropriate risk assessment framework, this study could be repeated in other locations, both nationally and internationally, to assess ETA risk in a consistent way. This study demonstrates how research can inform appropriate priority and resource allocation, especially in the public health sector.

## Figures and Tables

**Table 1:** International Classification of Disease (ICD)-coded asthma total presentations, daily presentation mean, maximum and number of case and control days for each hospital.

Hospital	Total Presentations	Daily Mean ^1^	Daily Maximum	No. of Case Days	No. of Control Days
Royal Hobart	7268	1.37	7	10	40
Launceston General	5807	1.09	8	16	64
MCH/NWRH ^2^ combined	6904	1.30	11	11	44

^1^ unadjusted daily mean; ^2^ Mersey Community Hospital/North West Regional Hospital.

## Supplementary Materials

The following are available online at https://www.mdpi.com/1660-4601/16/5/837/s1, Natural disaster risk assessment in Tasmania.
