# Supplementary material for: Evaluating the Risk of Epidemic Thunderstorm Asthma: Lessons from Australia
Source: Int J Environ Res Public Health. 2019 Mar 7;16(5):837. doi: 10.3390/ijerph16050837 (PMC6427665; doi:10.3390/ijerph16050837)
Supplement: Supplementary file 1 [file ijerph-16-00837-s001.pdf]

SUPPLEMENTARY MATERIAL for

Evaluating the risk of epidemic thunderstorm asthma: Lessons from Australia

Natural disaster risk assessment in Tasmania

The Tasmanian State Natural Disaster Risk Assessment (2016) (TSNDRA) uses five 'impact sectors' to determine the overall risk for each hazard. These sectors cover a range of consequences across a broad spectrum of outcomes, including:

1. People: Deaths or injuries as a direct consequence of the identified hazard.
2. Economic: The loss in economic activity or the economic impact on specific industries as a direct result of the identified hazard.
3. Environmental: The loss of ecosystems, species or environmental values as a direct result of the identified hazard.
4. Public administration: The decreased capacity of government and utilities to deliver core functions as a direct result of the identified hazard.
5. Social setting: The decreased capacity of the community to function as normal, or the loss of culturally significant objects or events as a direct result of the identified hazard [1,2].

For each sector, consequence categories determine the level of impact, rated from 'Catastrophic' to 'Insignificant' in the National Emergency Risk Assessment Guidelines (NERAG). For example, the 'People' sector defines a risk as 'Catastrophic' if more than 1 in 10,000 people are killed as a direct result of the event in a national context. For the Tasmanian context used in TSNDRA 2016, this equates to greater than 50 people.

As a specific type of risk, public health risk is largely determined by impact on the 'People' sector. Depending on the type of disaster, the public health risks can vary from fatalities as a direct result of the event, through to disruption of clean water supplies, disease outbreaks, and exacerbation of existing illnesses such as cardiovascular and respiratory illnesses [3]. However, the 'Social setting' sector, particularly an impact on the capacity of the community to function as normal, can have wide-ranging impacts on the health of community members, especially following a natural disaster or emergency [4].

Table S1. TSNDRA sectors and consequence categories assigned to ETA events in Tasmania.

Sector	Consequence category	Description of consequence category[1]	Evidence
People			
Death	Insignificant	Deaths directly from emergency >1 in 100,000,000 people (>0.005 people)	No ETA event occurred in Tasmania in the study period, therefore no deaths observed
Injury or Illness	Insignificant	Critical injuries with long-term or permanent incapacitation >1 in 100,000 people (>0.005 people)—OR—serious injuries >1 in 10,000 people (>0.5 people)	No ETA event occurred in Tasmania in the study period, therefore no injuries observed
Economic			
Activity/value	Insignificant	Economic decline and/or loss of asset value greater than 4% GSP (~$100k)	No evidence to suggest economic loss due to an ETA event
Impact on an important industry	Insignificant	Inconsequential business sector disruption due to emergency event	No evidence to suggest specific business or

			industry impact due to an ETA event
Environment			
Loss of species or landscape	Insignificant	Minor damage to an ecosystem or species recognized at the local or regional scale	No evidence to suggest landscape or species impact due to an ETA event
Loss of value	Insignificant	Inconsequential impact on environmental values of interest	No evidence to suggest impact on environmental values due to an ETA event
Public Administration	Insignificant	Governing bodies' delivery of core functions is unaffected or within normal parameters	While the Melbourne ETA event placed heavy demand on emergency services, [5,6] core government functions were not affected
Social Setting			
Loss of community wellbeing	Insignificant	The community of interest's social connectedness is disrupted such that the re-prioritization of existing resources is required, no dispersal	No evidence to suggest impact on social connectedness due to an ETA event. Some evidence of social connectedness increasing as a result of a natural disaster [7]
Loss of cultural significance	Insignificant	Minor damage to culturally significant objects – OR – minor delay of a major culturally important activity or event	No evidence to suggest impact on cultural objects or events due to an ETA event

[1] Descriptions taken from [1]

References

1. White, C.; Remenyi, T.; McEvoy, D.; Trundle, A.; Corney, S. *2016 Tasmanian State Natural Disaster Risk Assessment*; University of Tasmania, Hovart, Australia: 2016.
2. Commonwealth of Australia. *National Emergency Risk Assessment Guidelines – Handbook 10*; Australian Institute for Disaster Resilience, Attorney General's Department: Melbourne, Australia, 2015.
3. Noji, E.K. The Public Health Consequences of Disasters. *Prehospital Dis. Med.* **2000**, *15*, 21–31.
4. Commonwealth of Australia. *Australian Disaster Resilience Handbook 1: Disaster Health*; Australian Institute for Disaster Resilience, Attorney General's Department: 2011.
5. Victorian Government. The November 2016 Victorian epidemic thunderstorm asthma event: an assessment of the health impacts. Department of Health and Human Services: Melbourne, Australia, 2017.
6. Thien, F.; Beggs, P.J.; Csutoros, D.; Darvall, J.; Hew, M.; Davies, J.M.; Bardin, P.G.; Bannister, T.; Barnes, S.; Bellomo, R., et al. The Melbourne epidemic thunderstorm asthma event 2016: an investigation of environmental triggers, effect on health services, and patient risk factors. *Lancet Planet. Health* **2018**, *2*, e255-e263, doi:10.1016/S2542-5196(18)30120-7.
7. Thornley, L.; Ball, J.; Signal, L.; Lawson-Te Aho, K.; Rawson, E. Building community resilience: learning from the Canterbury earthquakes. *Kōtuitui: N. Z. J. Soc. Sci. Online* **2015**, *10*, 23-35, doi:10.1080/1177083X.2014.934846.
